# Current practice of family planning among teachers in public secondary school in Enugu East Senatorial District, Nigeria

**DOI:** 10.4314/ahs.v22i3.6

**Published:** 2022-09

**Authors:** Charles O Okafor, Nor Afiah Mohd Zulkefli, Sri Ganesh Muthiah

**Affiliations:** 1 Postgraduate Student in the Department of Community Health, Faculty of Medicine and Health Sciences, Universiti Putra Malaysia 43400 UPM Serdang, Selangor Darul Ehsan, Malaysia; 2 Department of Community Health, Faculty of Medicine and Health Sciences Universiti Putra Malaysia 43400 UPM Serdang, Selangor Darul Ehsan, Malaysia

**Keywords:** Teachers, Family Planning, Current Practice, Predictors, Nigeria

## Abstract

**Background:**

The aim of this study is to determine the current practice level of family planning and the associated factors among public secondary school teachers in Enugu East Senatorial District.

**Method:**

A cross-sectional study was carried out among public secondary school teachers, aged 18 - 60 years, in Enugu East Senatorial District, using probability proportional to size sampling and systematic random sampling to select 1000 participants. Binary and multiple logistic regression analyses were used to determine association. An odds ratio with a 95% confidence interval (CI) was computed to determine the level of significance.

**Results:**

The current practice level of family planning is 26.5%. Respondents with bachelor in education were 2 times more likely to be a current user of family planning (AOR=2.39; 95% CI: 1.25–4.55). However, respondents in age group 38 years and above were less likely to be a current user of family planning (AOR=0.64; 95% CI: 0.43–0.95), likewise female respondents (AOR=0.66; 95% CI: 0.44–0.98). Additionally, respondents who mentioned radio (AOR=0.64; 95%CI: 0.44–0.93), social media (AOR=0.73; 95% CI: 0.53–0.99) and healthcare (AOR=0.61; 95%CI: 0.43–0.88) as source of information were less likely to be current user of family planning. Whereas, partner who encouraged the use of family planning (AOR=2.54; 95% CI: 1.71–3.78) span style="font-family:'Times New Roman'; font-weight:bold">, partner who allow each other to decide on family planning methods (AOR=4.47; 95% CI: 2.67–7.48) and those who had good knowledge of family planning (AOR=1.96; 95% CI: 1.40–2.67) were more likely to be current user of family planning.

**Conclusion:**

The level of current practice of family planning is low and a significant number of factors predict the current practice of family planning. A family planning educational workshop among teachers is needed to improve teacher's knowledge on family planning to address the issue of adolescent sexual reproduction as teachers are vessels of knowledge impartation to students.

## Introduction

Family planning is a process that allows individuals and couples to attain their desired number of children. The spacing and timing of their births are achievable through the use of contraceptive methods and the treatment of family planning inadequacies according to the World Health Organization^1^. In developing nations, family planning inadequacies are the major contributing factor to poverty, ill health, poor economic development and political instability. Adequate family planning utilization could save approximately $5.7 billion by preventing unplanned pregnancies and unsafe abortions^2^,^3^. Population dynamics and the adverse effects have led to the adaptation of family planning as a tool of a global master plan to reduce population growth, which benefits the health of women and children^4^. It also contributes towards the achievement of the sustainable development goals (SDGs) and the target of the “health for all” policy^5^.

Nigeria has over 35 million women of reproductive age, with 7 million births occurring annually. Over 40% of Nigeria's population are young people with the median age for first time sexual intercourse being 17.9 years^6^,^7^. Teenagers account for 60% of the estimated 600,000 induced abortions annually and for every 1000 teenage pregnancies in Nigeria, 250 ends in unsafe abortions, contributing to a high maternal mortality^8^. More importantly, the maternal mortality ratio of Enugu State was estimated at 840/100 000 live births, higher than the national average of 545/100 000 live births, due to poor utilization of contraceptives. The national contraceptive prevalence rate in 2018 was 15% compared to South Africa, USA and UK with a contraceptive prevalence rate of 55%, 74% and 84%, respectively^9^–^13^. Apart from obstetric complications experienced in teenage pregnancy, there are other consequences such as termination of academic pursuits, low job opportunities, isolation, loss of self-esteem and repeat pregnancy^14^. Many factors have been found to influence current practice of family planning around the world. These factors have both positive and negative impact on current practice of family planning, which include: socio demographic characteristics (age, gender, ethnicity, religion, evel of education, partner's level of education, occupation, monthly income, marital status, marriage type, marriage duration, parity and place of residence), sources of information (television, radio, social media, newspaper, friends and relatives), partner involvement (partner discussion, partner encouragement, partner support, partner approval and partner decision). This has necessitated this study to assess the current practice of family planning among public secondary school teachers because teachers are the vessels for knowledge impartation to students, to enhance learning and understanding. Moreover, teacher's knowledge and experience on family planning will improve the quality of student's relationships and their ability to make informed decision over their life time^15^. No study of this nature has ever been conducted among teachers in Enugu East Senatorial District. The findings of the study will contribute to the existing literature that will help the policymakers of the district, at regional and national levels, in making pragmatic policies to improve the teachers' knowledge and enhance their teaching ability regarding adolescent sexual and reproductive health, by creating programs that pave way for easy communication between teachers and students as the youth are known as the future of tomorrow.

## Methodology

### Study Design

This study was conducted using a cross-sectional study design between August-October 2019 in the Enugu East Senatorial District, Nigeria. The District comprise six local government areas, namely: Isi Uzo, Enugu East, Enugu North, Enugu South, Nkanu West and Nkanu East, which are located on the eastside on the Enugu State, southeast Nigeria with a total land area of 3,046km^2^ and an estimated population of 1,166,944^16^. The district also has 82 public secondary schools with 3,236 teachers out of 11,873 teachers in the entire state^17^.

### Sample size and Sampling Technique

The sample size was calculated using two proportion (two-sided test) Lemeshow formula with a requirement for significance level (α) of 0.05 and 90% power with the assumption of a 5% margin of error, 95% confidence level^18^; a total of 1000 sample size was estimated. This study applied probability proportional to size sampling to determine the total number of participants in each local government by sampling the entire list of six local governments in the senatorial district together with a total number of 3,236 teachers. Systematic random sampling, k=population size/sample size, was then used to select the actual participants in this study. Inclusion criteria; all teachers both male and female within civil service age (18–60 years)^19^, married, single, and those who have reached menopause that were using sterilization and Long acting and permanent contraceptive methods before menopause, and those who utilize family planning methods for protection against sexual transmitted infection^20^,^21^, that are currently teaching in a public secondary school in Enugu East Senatorial District were selected. Exclusion criteria; all teachers who were not present or refuse to take part in the study and those who were pregnant during the data collection were excluded from this study.

### Data Collection Technique and Quality Control

The outcome variable analysed in this study is current practice of family planning and is defined as currently using either modern or traditional method of family planning within the period of data collection of this study^22^. A pretested self-administered structured questionnaire was used for data collection which was adapted from previous studies^23^–^27^. The reliability and validity of the instrument was checked and all the questions have a Cronbach's alpha of 0.7. The questionnaire was divided into six sections (A-F). Section (A) was a socio-demographic questionnaire consist of age, gender, marital status, ethnicity, educational level, occupation, monthly income, religious belief, place of residence and parent's education, type of family, duration of marriage and parity^25^,^28^,^29^. Section (B) was knowledge on family planning with 25 items related to knowledge of family planning^30^. Participants had the option of selecting Yes, No or Don't know in response to the questions. Each correct response was scored 1 point, whereas a wrong or don't know response scored 0 points, with the total being 25 points. The knowledge level was categorized into good or poor knowledge based on the median cut-off score. A score of 12 and above was considered a good level of knowledge on family planning. Section (C) focused on attitudes towards family planning^24^,^31^ with 10 items that related to attitudes towards family planning and was measured depending on the degree of agreement with the expressed sentiment on a five-point Likert scales, ranging from strongly disagree, disagree, not sure, agree and strongly agree. Their responses were graded as follows 1, 2, 3, 4, and 5 respectively, with the minimum score of 1 designated “strongly disagree” and the maximum score of 5 designated “strongly agree”. The total minimum score obtained was 10 while the total maximum score obtained was 50. Attitude level was categorized into positive and negative attitudes, based on the median cut-off mark and those who scored 26 and above, were classified as having a positive attitude towards family planning. Section (D) looked at sources of information; namely; hospital/healthcare facilities, social media, television, radio, newspaper, parent/guardian, teachers, friends, book, church, mosque and other sources not mentioned^25^. Section (E) was a partner involvement questionnaire with five items related to the husband and wife's level of relationship and their involvement in family planning^27^,^29^. Each question had an option of Yes, No and Not sure for answers. Yes was coded as “1” while No and Not sure was coded as “0”. Section (F) was a current practice questionnaire with four items which were relevant to the practice of family planning which include modern and traditional methods of family planning^32^. The questions provide aoption of Yes or No answers and a specific answer for the family planning method currently used by the respondent and reason for not practicing family planning, Yes was coded as “1” while No was coded as “0”. A consent form was sent to the respondents to obtain their permission to participate in the study and verbal permission to carry out the data collection was obtained from the principal of the various schools. All respondents were informed that they had the right to withdraw from this study at any time. The respondents were assured that their information would be kept private and confidential at all times. This study was approved by the Ethics Committee involving Human Research of the Universiti Putra Malaysia (JKEUPM-2010-138). Further permission to conduct this study was also obtained from the Enugu State Ministry of Education, Nigeria.

### Statistical Analysis

Data was analysed using IBM SPSS software version 25, data entry and data transformation. An investigation was carried out and conveyed as follows; a normality test was done to check the distribution of the continuous data, however, the data was not normally distributed, so the result was presented with median and interquartile ranges (IQR) and then converted into categorical data. Descriptive statistics (frequency and percentage) were used to describe categorical variables such as socio-demographic characteristics, sources of information, partner involvement history of family planning methods, healthcare facility and current practices of family planning. Binary logistic regressions were used to test for predictors of current practice of family planning. All the significant independent variables in bivariate analysis at p-value <0.25 were analysed in the multivariate analysis to identify independent predictors using both forward and backward conditional methods^33^. A significant list of the best predictors was obtained at P value less than 0.05 and 95% confidence interval in the final model.

## Results

### Socio-demographic Characteristics

One thousand teachers were recruited in this study, yielding a 100% response rate. More than half (51.3%) of the respondents were below 38 years old, and the vast majority of respondents (71.5%) were female. Almost all of them were Igbos ethnic group (92.2%). Christianity constituted the major (98.6%) religion. The distribution of the participant's level of education showed that more than half of the respondents (53.0%) had a bachelor's in education. In contrast, the partner's level of education was mainly (76.3%) those that possess B.Sc., M.Sc., and Ph.D. The majority (60.1%) of respondents were non-science teachers, while almost all the respondents (85.4%) received a monthly salary of NGN27, 000 naira and above. Additionally, most of the respondents (75.2%) were married, and almost all (92.3%) of them were in a monogamous type of marriage. However, more than half (51.8%) of respondents were married for less than 12 years. The majority of respondents (71.5%) reside in an urban area, while more than half (62.1%) of the respondents had 3 and above children, as shown in table 1.

**Table 1:** Distribution of Respondents by Socio-demographic Characteristic (N=1000)

Socio-demographic Characteristics	n	%	Median (IQR
**Age groups (years)**			38(17)
< 38	513	51.3	
≥ 38	487	48.7	
**Gender**			
Male	285	28.5	
Female	715	71.5	
**Ethnicity**			
Igbo	922	92.2	
Others	78	7.8	
**Religion**			
Christian	986	98.6	
Others	14	1.4	
**Level of education**			
Nigerian Certificate in Education	96	9.6	
Bachelor in Education (B.Eds)	530	53.0	
Postgraduate Diploma in Education	169	16.9	
Other Qualification	205	20.5	
**Partners levels of Education (992)**			
First Living Certificate	47	4.7	
WAEC/NECO	143	14.3	
BSc, MSc, PhD	763	76.3	
No educational qualification	47	4.7	
**Occupation**			
Science Teachers	399	39.9	
Non Science teachers	601	60.1	
**Monthly Income (Naira)**			
< N27, 000	146	14.5	
≥ N27, 000	854	85.4	
**Marital status**			
Single	209	20.9	
Married	752	75.2	
Divorced	13	1.3	
Widowers	26	2.6	
**Marriage Type (791)**			
Monogamous	930	92.3	
Polygamous	61	7.7	
**Marriage Duration (791)**			12(13)
< 12 years	410	51.8	
≥ 12 year	381	48.2	
**Place of Resident**			
Urban	715	71.5	
Rural	285	28.5	
Parity (791)			
< 3 children	300	37.9	3(2)
≥ 3 children	491	62.1	

### Sources of Information on Family Planning

Table 2 showed the distribution of respondents by sources of information on family planning. Majority of the respondent cited healthcare facilities (63.6%), followed by television (60.4%) and radio (54.2%). However religious institution (Church) provided the least sources of information (11.0%).

**Table 2:** Distribution of Sources of Information on Family Planning (N=1000)

Variables	n	%
Television		
No	396	39.6
Yes	604	60.4
Radio		
No	458	45.8
Yes	542	54.2
Social Media		
No	600	60.0
Yes	400	40.0
Newspapers		
No	341	34.1
Yes	659	65.9
Books		
No	737	0.73.7
Yes	263	26.3
Healthcare		
No	364	36.4
Yes	636	63.6
Parents and guardian		
No	757	75.7
Yes	243	24.3
Teachers		
No	716	71.6
Yes	284	28.4
Friends		
No	591	59.1
Yes	409	40.9
Church		
No	890	89.0
Yes	110	11.0

### Partner Involvement in Family Planning

The majority of the respondents (67.4 %) discussed family planning with their partners, followed by those who approve the use of family planning methods with their partner (62.7%). While partner's decision (56.6%), partner encouragement (49.4%), and partner support (48.7%), provides the least of partner involvement on family planning. However, majority (61.3%) of respondents had a good level of knowledge on family planning and more than half of the respondents (52.7%) were found to have positive attitudes towards family planning as shown in table 3.

**Table 3:** Partner Involvement on Family Planning by the Respondents (N=1000)

Variables	n	%	Median (IQR)
Partner's discussion			
No	326	32.6	
Yes	674	67.4	
Partner encouragement			
No	506	50.6	
Yes	494	49.4	
Partner's approval			
No	373	37.3	
Yes	627	62.7	
Partners support			
No	513	51.3	
Yes	487	48.7	
Partner's decision			
No	433	43.3	
Yes	567	56.7	
Levels of knowledge			12(5)
Good	61.3		
Poor	387	38.7	
Levels of attitudes			26(10)
Positive	527	52.7	
Negative	473	47.3	

### Current Practice of Family planning

In this study, about 26.5% of respondents were currently practicing family planning methods, while only 17.4% of respondent's partners were using family planning methods simultaneously. Almost all the male respondents were using condom (81.2%), followed by withdrawal methods (16.7%) and the least methods are vasectomy (1.2%). In contrast, majority of female respondents were using contraceptive implant/implanon (36.1%) followed by calendar or rhythm methods (16.9%) and the least used methods among the female respondents was intrauterine device (IUD) (2.7%) in Table 4.

**Table 4:** Current Practice of Family Planning by Respondents

Variables	n	%
Current practice		
No	753	75.3
Yes	265	26.5
Partner current practice		
No	826	82.6
Yes	174	17.4
Methods currently using **(male, n=84)**		
Condom	69	81.2
Withdraw methods	14	16.7
Sterilization (Vasectomy)	1	1.2
**(Female, n=181)**		
Contraceptive implants / implano	66	36.1
Calendar Methods	31	16.9
Billing ovulation Methods	20	10.9
Oral contraceptive pill	17	9.3
Injectable/ depoprovera	16	8.7
Condom	12	6.7
Sterilization (Tubal ligation)	8	4.4
Emergency contraceptive pill	6	3.3
Intrauterine device (IUD)	5	2.7

### Reason for not Practicing Family Planning Method

Most respondent have no reason not practicing family planning (28.8%), followed by those who were still bearing children (21.8%), and the least was being afraid of the side effect (1.8%).

### Socio-Demographic Characteristics Associated with Current Practice of Family planning

Simple and multiple logistic regressions were fitted to identify socio-demographic predictors of the current practice of family planning. However, the age group of below 38 years old has higher odds of practicing family planning compared to the age group of 38 years and above (AOR=0.64; 95%CI: 0.43–0.95). Male respondents has higher odds of practicing of family planning than their female counterparts (AOR=0.66; 95%CI: 0.44–0.98) as presented in Table 5.

**Table 5:** Socio-Demographic Determinant of Current Practice of Family Planning among Public Secondary School Teachers in Enugu East Senatorial Districts (n=1000)

Variable	Current Practice of Family Planning		95%CI	
	Yes	No		
	n(%)	n(%)	COR	AOR
Age group				
< 38	119(23.2)	394(76.8)	1	1
≥ 38	146(30.0)	341(70.0)	0.66(0.39–1.12)	0.67(0.43–0.95)*
Gender				
Male	84(29.5)	201(70.5)	1	1
Female	181(25.3)	534(74.7)	0.65(0.42–1.10)	0.66(0.44–0.98)*
Ethnicity				
Igbo	253(27.4)	669(72.6)	1	1
Others	12(15.4)	66(84.6)	0.38(0.13–1.14)	0.41(0.14–1.18)
Religion				
Christianity	262(26.6)	724(73.4)	1	
Others	03(21.4)	11(78.6)	0.75(0.21–2.73)	
Level of education				
National Certificate Edu.	22(22.9)	74(77.1)	1	1
Bachelor in education	161(30.4)	369(69.6)	2.32(1.20–4.49)*	2.39(1.25–4.55)**
Postgrad. Diploma edu.	46(27.2)	123(73.8)	2.23(1.06–4.72)*	2.37(1.14–4.91)*
Others qualification	36(17.6)	169(83.4)	1.31(0.58–2.95)	1.34(0.60–2.98)
Partner's education				
First living certificate	17(37.0)	29(63.0)	1	1
WAEC/NECO	54(37.8)	89(62.2)	0.62(0.25–1.54)	0.63(0.26–1.55)
BSc, MSc, &PhD	173(22.9)	584(77.1)	0.23(0.09–0.54) **	0.23(0.10–0.53)**
No Education	20(43.5)	26(56.5)	0.67(0.24–2.02)	0.69(0.23–1.85)
Occupation				
Science teachers	120(30.1)	279(69.9)	1	
Non- sci. teachers	145(24.1)	456(75.9)	0.74(0.56–0.98)*	
Monthly income				
< NGN27, 000	33(22.6)	113(77.4)	1	
≥ NGN27, 000	232(27.2)	622(72.8)	1.28(0.84–1.94)	
Marital status				
Single	32(15.3)	177(84.7)	1	
Married	223(29.7)	529(70.3)	2.33(1.55–3.51)***	
Divorced	4(30.8)	9(69.2)	2.46(0.71–8.47)	
Widowers	6(23.1)	20(76.9)	1.66(0.62–4.45)	
Type of marriage				
Monogamous	209(28.6)	523(72.4)	1	
Polygamous	24(39.3)	37(60.7)	1.62(0.95–2.78)	
Marriage Duration				
< 12 years	120(29.3)	290(70.7)	1	
≥ 12 years	145(24.6)	445(76.4)	0.61(0.36–1.05)	
Place of residents				
Urban	192(26.9)	523(74.1)	1	
Rural	73(25.6)	212(75.4)	1.07(0.78–1.46)	
Parity (Children)				
< 3	78(15.4)	430(85.4)	1	1
≥ 3	187(38.0)	305(62.0)	3.39(2.55–6.00) ***	4.08(2.68–6.23)***

Note (*)-significance level (p)<0.05, **p<0.01 and ***p<0.001, CI= confidence interval, COR= Crude Odd ratio, AOR= Adjusted Odd ratio, Reference Category= 1

### Other Factors Associated with Current Practice of Family Planning

Table 6 illustrate the result of simple and multiple logistic regression analysis which revealed the predictors of current practice of family planning which includes: radio (AOR=0.64; 95%CI: 0.44–0.93), social media (AOR=0.64; 95%CI: 0.44–0.95), healthcare (AOR=0.61; 95%CI: 0.43–0.88), partner's encouragement (AOR=2.56; 95%CI: 1.63–4.03), partner's support (AOR=1.64; 95%CI: 1.04–2.59), partner's decision (AOR=7.41; 95%CI: 4.45–12.33), having good knowledge of family planning (AOR=1.70; 95%CI: 1.20–2.42) and having positive attitude (AOR=1.59; 95%CI: 1.14–2.24).

**Table 6:** Other Factors of Current Practice of Family Planning among Public Secondary School Teachers in Enugu East Senatorial Districts (n=1000)

Variable	Current Practice			
	Yes	No	COR	AOR
	N (%)	n(%)	(95%CI)	(95%CI)
Television				
No	102(25.8)	294(74.2)	1	
Yes	163(27.0)	441(73.0)	1.16(0.72–1.86)	
Radio				
No	126(27.5)	332(72.5)	1	1
Yes	139(25.6)	403(74.4)	0.57(0.34–0.96)*	0.64(0.44–0.95)*
Social media				
No	165(27.5)	435(72.5)	1	1
Yes	100(25.0)	300(75.0)	0.58(0.38–0.87)**	0.64(0.44–0.93)*
Newspapers				
No	173(26.3)	486(73.7)	1	
Yes	92(27.0)	249(73.0)	0.99(0.64–1.62)	
Books				
No	197(26.7)	540(73.3)	1	
Yes	68(25.9)	195(74.1)	0.76(0.49–1.16)	
Healthcare				
No	89(24.5)	275(75.5)	1	1
Yes	176(27.7)	460(72.3)	0.58(0.38–0.87)**	0.61(0.43–0.88)**
Parent or guardian				
No	196(25.9)	561(74.1)	1	
Yes	69(28.4)	174(71.6)	098(0.63–1.51)	
Teachers				
No	170(23.7)	546(76.3)	1	
Yes	95(33.5)	189(66.5)	1.31(0.90–1.90)	
Friends				
No	134(22.7)	456(77.3)	1	
Yes	131(32.0)	276(68.0)	1.29(0.89–1.87)	
Church				
No	232(26.1)	405(45.5)	1	
Yes	65(59.1)	45(40.9)	1.53(0.83–2.80)	
Partner's discussion				
No	16(4.9)	310(95.1)	1	
Yes	249(36.9)	425(63.1)	1.56(0.61–3.98)	
Partners encourage				
No	53(10.5)	453(89.1)	1	1
Yes	212(42.9)	282(57.1)	2.34(1.43–3.83)**	2.56(1.63–4.03)***
Partner's approval				
No	23(6.2)	350(93.8)	1	
Yes	242(38.6)	385(61.4)	0.84(0.35–2.05)	
Partner's support				
No	60(11.7)	453(88.3)	1	1
Yes	205(42.1)	282(57.9)	1.64(1.008–2.69)*	1.65(1.04–2.59)*
Partner's decision				
No	28(6.5)	405(93.5)	1	1
Yes	237(41.8)	330(58.2)	6.67(3.55–12.56)***	7.41(4.45–12.33)***
Levels of knowledge				
Poor	73(18.9)	314(81.1)	1	1
Good	192(31.3)	421(68.7)	1.65(1.46–2.37)***	1.70(1.20–2.42)**
Levels of attitudes				
Negative	123(26.0)	350(74.0)	1	1
Positive	142(26.9)	385(73.1)	1.52(1.07–2.16)*	1.59(1.14–2.24)**

Note (*)-significance level (p)<0.05, **p<0.01 and ***p<0.001, CI= confidence interval, COR= Crude Odd ratio, AOR= Adjusted Odd ratio, Reference Category= 1

## Discussion

In this study, we found a low level of current practice of family planning among the respondents (26.5%), despite the moderate levels of knowledge and attitudes of the respondents towards family planning. A possible explanation to this finding may be lack of awareness as our result show that most respondents who obtained information on family planning from these relevant channels were less likely to practice family planning. This result is higher than the Nigerian national average contraceptive prevalence rate of 15%(7). However, this current finding contradicts Nigeria and Uganda's findings, which revealed a much higher percentage of current use of family planning of 35.2% and 38%, respectively^34^,^35^. The discrepancies in these studies are mostly as a result of experienced gained during antennal and postnatal care because most of the respondents in these previous studies were married and had at least a child^36^. One of the major reasons for not practicing family planning among the respondents in this study is the desire to have more children. This study also found that most male respondents were either using a condom (81.2%) or withdrawal method (16.7%). The least used method was having a vasectomy (1.2%). In comparison with female respondents who were mostly using contraceptive implants (36.1%), followed by a calendar method (16.9%) and the least used method among them was an intrauterine device (IUD) (2.7%). Most of them had no reason for not practicing family planning (28.8%) and about 21.8% of them were still having children, with a few (1.8%) being afraid of the possible side effects (1.8%). This is consistent with findings in Nigeria among women of reproductive age, which revealed that condoms, rhythm and pill were the most widely used contraceptives. Simultaneously, the reasons for not practicing included – wanting more children, afraid of side effects and pregnancy^34^.

This study also found that age group below 38 years old, male respondents, those with national certificate in education (NCE), bachelor in education (B.Ed) as well partners with first living certificate and respondents with three children and above had higher odds of adopting a current family planning practice as compared to other counterparts. The rationale behind these findings is good knwledge of family planning found in this study. This is supported by the findings in Nigeria and elsewhere. Furthermore, the findings of a study among rural women in kebeles of Dembia District Ethiopia revealed that respondents below 35 years of age were almost twice as likely to be current users of modern contraception as those above 35 years of age because they want to space their child births because most them were married already with a child. The findings in Uganda among HIV patients also revealed that male respondents were more likely to be current users of family planning than their male counterparts but not statistically significant^29^,^32^,^35^.

Our study also found that those who cited radio, social media and healthcare had lower odds of adopting a current family planning practice than those who did not cite either radio, social media or healthcare. This is probably due to most respondents preferring to seek counsel on matters relating to their sexual reproductive health from superiors, for example, teachers and friends, also due to the fact that social media is new in our society. In addition, more awareness on the specific methods of family planning and the benefit are needed to improve family planning utilization. Similar findings were reported in Ghana and the United States of America^37^,^38^. Moreover, the content of messages delivered through these channels should be short and simple to facilitate easy understanding.

Our study's findings also show that encouragement from the respondent's partner, support of partners and freedom to decide on family planning methods showed a higher chance of adopting a current family planning practice than other counterparts. This could be attributed to the cordial relationship existing between the partners and evident of good knowledge and positive attitudes towards family planning found among respondents in this study. These findings are supported by studies in Ethiopia, Ghana and Palestine, which revealed that partners involved in family planning either by mutual communication, encouragement or decision making on family planning methods increase the current utilization of family planning methods^27^,^29^,^32^,^39^,^40^. This study revealed that respondents with good knowledge and positive attitude towards family planning had higher odds of practicing family planning than those with poor knowledge and negative attitudes towards family planning. A plausible explanation is the high literacy level found among the respondents in this study. Additionally, family planning educational workshop is needed to improve teacher's knowledge on family planning benefit; side effect and family planning methods utilization and the knowledge acquired from this study will help the teachers to address the issue of adolescent sexual reproductive health. This is consistent with the findings in Ethiopia^40^,^41^. Limitation of this study includes, the cross sectional study, so causality cannot be established. Also, due to the volume of questions, most of the respondents submitted incomplete questionnaires. The study's findings are based on the responses of the participants which may be subject to some levels of response bias.

## Conclusion

In this study, the percentage of current practice of family planning among the teachers at public secondary schools in Enugu East Senatorial District is very low with the most preferred method of family planning among male respondents being the use of a condom, while contraceptive implant is the most used method among female respondents. Most of the respondents have no reason for not being a current user of family planning methods. In most cases, the current practice of family planning was predicted by some significant factors. Furthermore, this study recommends a family planning educational workshop among the teachers in the districts to help create awareness on family planning and train teachers to improve teacher's knowledge on family planning to address the issue of adolescent sexual reproductive health as teachers are vessels of knowledge impartation to students especially the adolescents group. This can strengthen the current school-based program. Further research should be conducted using case control or cohort study to establish causal relationship. Mixed method study should be conducted to explore more on the predictors in depth as well as interventional study from the findings able to develop health education and study the effectiveness.

## References

[1] Health R, Bank W, Programme S, Reproduction H (2009). RHR Highlights 2008 Highlights of 2008 RHR Highlights 2008.

[2] Darroch JE, Singh S, Nadeau J (2008). Contraception: an investment in lives, health and development. Issues Brief (Alan Guttmacher Inst).

[3] Bremner J, Frost A, Haub C, Mather M, Ringheim K, Zuehlke E (2010). World Population Highlights :Population Reference Bureau. Popul Bull.

[4] UNFPA (2014). Population and sustainable development in the Post-2015 agenda.

[5] World Health Organization Department of Reproductive Health Services (2015). Improving Access to Quality Care in Family Planning: Medical Eligibility Criteria for Contraceptive Use. Eff Br mindfulness Interv acute pain. Exp An Exam Individ Differ.

[6] United Nations (2013). World Population Prospects: The 2012 Revision. Highlights and Advance Tables. Popul Dev Rev.

[7] National Population Commission of Nigeria (2014). Nigeria Demographic and Health Survey 2013.

[8] Isonguyo IN (2013). Adolescents and Utilization of Family Planning Services in Rural Community of Nigeria. Res Humanit Soc Sci.

[9] NDHS NDHS. National Population Commission (NPC). ICF International (2013). Nigerian Demographic and Health Survey 2013.

[10] Kaneda T, Dupuis G, Bietsch K (2015). 2015 World Population Data Sheet.

[11] Eze P, Ezenduka C, Obikeze E, Ogbuabor D, Arize I, Ezenwaka U (2020). Examining the distribution of benefits of a free Maternal and Child Health programme in Enugu State, Nigeria: a benefit incidence analysis. Trop Med Int Heal.

[12] Africa SS (2017). South Africa demographic and health survey 2016: Key indicator report.

[13] Hoque M (2017). The way ahead. Biotechnol Sustain Agric Emerg Approaches Strateg.

[14] Ezegwui HU, Ikeako LC, Ogbuefi F (2012). Obstetric outcome of teenage pregnancies at a tertiary hospital in Enugu, Nigeria. Niger J Clin Pract.

[15] Adogu POU, Nwafulume OS (2015). Knowledge, Attitude and Willingness to Teach Sexuality Education among Secondary School Teachers in Nnewi, Nigeria.

[16] NPC (2007). Report of Nigeria's National Population Commission on the 2006 census.

[17] Enugu State Ministry of Education (2014). Annual School Census Report.

[18] Ogston SA, Lemeshow S, Hosmer DW, Klar J, Lwanga SK (1991). Adequacy of Sample Size in Health Studies. Biometrics.

[19] Federal Republic of Nigeria (2005). Action Programme on Education, 2004-2005 Teachers for the Future: Meeting Teacher Shortages To Achieve Education for All National Policy Brief, Nigeria.

[20] Schwandt HM, Skinner J, Hebert LE, Cobb L, Saad A, Odeku M (2017). Inadequate birth spacing is perceived as riskier than all family planning methods, except sterilization and abortion, in a qualitative study among urban Nigerians. BMC Womens Health.

[21] Abdisa B, Mideksa L (2017). Factors Associated with Utilization of Long Acting and Permanent Contraceptive Methods among Women of Reproductive Age Group in Jigjiga Town. Anat Physiol.

[22] Adedini Sunday A, Babalola Stella, Ibeawuchi Charity, Omotoso Olukunle, Akiode Akinsewa, MO (2015). Role of Religious Leaders in Promoting Contraceptive Use in Nigeria : Evidence From the Nigerian Urban Reproductive Health Initiative.

[23] JA A, PE I (2016). Reproductive health knowledge and sexual behaviours of adolescents with learning disabilities in Ibadan North local government area, Oyo State. Educ Res.

[24] Omishakin MYJ (2015). Women ‘ s Health & Gynecology Knowledge , Attitude and Practice of Family Planning among Healthcare Providers in Two Selected Health Centres in Osogbo Local Government , Osun State. Womens Heal Gynecol.

[25] Bugssa G (2014). Factors Associated with Knowledge, Attitude and Practice towards Emergency Contraception among Female Clients of Ethiopian Immigration and Nationality Affairs Office. J Community Med Health Educ.

[26] Sherpa SZ, Sheilini M, Nayak A (2013). Knowledge, attitude, practice and preferences of contraceptive methods in udupi district, karnataka. J Fam Reprod Heal [Internet].

[27] Kassa M, Abajobir AA, Gedefaw M (2014). Level of male involvement and associated factors in family planning services utilization among married men in Debremarkos town, Northwest Ethiopia. BMC Int Health Hum Rights.

[28] Yu C, Chen R, Li JJ, Li JJ, Drahansky M, Paridah M (2012). We are IntechOpen , the world ‘ s leading publisher of Open Access books Built by scientists , for scientists TOP 1 %. Intech [Internet].

[29] Eliason S, Awoonor-Williams JK, Eliason C, Novignon J, Nonvignon J, Aikins M (2014). Determinants of modern family planning use among women of reproductive age in the Nkwanta district of Ghana: A case-control study. Reprod Health.

[30] Chimah UC, Lawoyin TO, Ilika AL, Nnebue CC (2016). Contraceptive knowledge and practice among senior secondary schools students in military barracks in Nigeria. Niger J Clin Pract.

[31] Owolabi EO, Goon D Ter, Seekoe E (2017). Attitude to, and knowledge and practice of family planning among women of child-bearing age attending selected hospitals in Osogbo, Nigeria. Afr J Nurs Midwifery.

[32] Debebe S, Limenih MA, Biadgo B (2017). Modern contraceptive methods utilization and associated factors among reproductive aged women in rural Dembia District, northwest Ethiopia: Community based cross-sectional study. Int J Reprod Biomed.

[33] Hosmer DW, Lemeshow S (2000). Applied logistic regression.

[34] Duru CB, Emelumadu OF, Iwu AC, Ohanle I, Agunwa CC, Nwaigbo E (2018). Socio-Demographic Determinants of Family Planning Service Utilization among Women of Reproductive Age in Urban Communities of Imo State, Nigeria. OALib.

[35] Nattabi B, Li J, Thompson SC, Orach CG, Earnest J (2011). Family planning among people living with HIV in post-conflict Northern Uganda: A mixed methods study. Confl Health [Internet].

[36] Mansor MB, Abdullah KL, Oo SS, Akhtar K, Jusoh ASB, Ghazali SB (2015). The prevalence of family planning practice and associated factors among women in Serdang, Selangor. Malaysian J Public Heal Med.

[37] Hodogbe EA, Badu-Nyarko SK (2015). Knowledge and Practice of Family Planning among Female Basic School Teachers in the City of Accra, Ghana. Int J Soc Sci Stud.

[38] Bekalu MA, McCloud RF, Viswanath K (2019). Association of Social Media Use With Social Well-Being, Positive Mental Health, and Self-Rated Health: Disentangling Routine Use From Emotional Connection to Use. Heal Educ Behav.

[39] Hammoudeh WS, Health- P (2009). Factors associated with the use of family planning among Palestinian women.

[40] Tesfa E, Gedamu H (2018). Factors associated with utilization of long term family planning methods among women of reproductive age attending Bahir Dar health facilities, Northwest Ethiopia. BMC Res Notes [Internet].

[41] Alemayehu M, Lemma H, Abrha K, Adama Y, Fisseha G, Yebyo H (2016). Family planning use and associated factors among pastoralist community of afar region, eastern Ethiopia. BMC Womens Health [Internet].

